# Maternal and perinatal outcomes across China's one-child, two-child, and three-child policy periods: a retrospective cohort analysis of 43,439 deliveries

**DOI:** 10.7189/jogh.16.04328

**Published:** 2026-09-18

**Authors:** Haijuan Li, Dongmei Tang, Dan Yang

**Affiliations:** Department of Obstetrics and Gynecology, Wuming Hospital of Guangxi Medical University, Nanning, China

**Keywords:** fertility policy, maternal health, perinatal outcomes, advanced maternal age pregnancy, caesarean section, pregnancy complications

## Abstract

**Background:**

China has sequentially implemented the one-child, two-child and three-child policies over the past 15 years, yet long-term effects of these fertility policy adjustments on maternal and perinatal health remain understudied. This study aimed to evaluate such impacts.

**Methods:**

A single-centre retrospective cohort analysis was conducted on 43,439 parturients delivering at a Guangxi hospital between 2011 and 2025. All participants were grouped into one-child (OCP), two-child (TCP) and universal three-child (UCP) policy cohorts. Multivariate logistic regression was applied to adjust for confounders and analyse the associations between fertility policies and the risks of caesarean section and postpartum haemorrhage (PPH).

**Results:**

Policy relaxation significantly increased maternal mean age (27.58 ± 4.85 to 31.37 ± 5.57 years), advanced maternal age (AMA) proportion (≥35 years, 9.3–30.5%), and *in vitro* fertilisation (IVF) utilisation (1.3–6.2%), while reducing the primipara ratio (66.3–39.9%) (all *P* < 0.001). Gestational diabetes mellitus (GDM) prevalence reached 26.7%, placental abruption (0.3–1.6%), caesarean section rate (29.6–39.9%), and emergency admissions (11.9–45.3%) all increased; hospital stay decreased (5.82–4.73 days) (all *P* < 0.001). Multivariate analysis showed lower caesarean section risk in TCP (adjusted odds ratio (aOR) = 0.91; 95% confidence interval (CI) = 0.86–0.95) but higher in UCP (aOR = 1.08; 95% CI = 1.02–1.15); an increase of one year in age raised caesarean risk by 8% (aOR = 1.08; 95% CI = 1.07–1.09). The TCP period exhibited the highest risk of PPH (aOR = 1.80; 95% CI = 1.55–2.10). Though caesarean delivery correlated with diminished PPH risk (aOR = 0.28; 95% CI = 0.24–0.33), this correlation stemmed from discrepant PPH diagnostic thresholds by delivery mode rather than a genuine protective benefit.

**Conclusions:**

Fertility policy relaxation coincided with altered maternal demographic profiles, elevated high-risk pregnancy and obstetric complication burdens, and greater emergency care demand. Such correlations highlight an urgent need for targeted optimisation of maternal healthcare delivery systems.

Population policies are powerful, deliberately constructed institutional interventions that profoundly reshape a nation's social, familial, health, and demographic structure [1]. Over the past ten years, China's fertility policy has changed. The long-standing one-child policy (OCP) was abandoned in favour of the two-child policy (TCP) in 2016 [2], and the three-child policy was finally authorised in 2021 [3]. These changes have been driven by a rapidly aging population, a shrinking workforce, and a declining birth rate [4]. The core objective of this series of policy adjustments is to boost fertility, optimise the demographic structure, and sustain economic growth. However, the short-term impacts and long-term cumulative effects of policy relaxation on maternal and neonatal health are yet to be systematically verified through large-scale real-world data that includes the full policy cycle. This gap has created the need for precise scientific evaluations to investigate its impact on public health decision-making.

Currently, studies show that the implementation of the TCP considerably reshaped obstetric clinical practice. It resulted in a significant influx of older multiparous women, specifically those of advanced maternal age (AMA), aged ≥35 years per clinical diagnostic criteria adopted in this research [5]. This directly elevated the risk of complications during pregnancy, such as hypertensive disorders of pregnancy (HDP, which includes gestational hypertension, preeclampsia, and eclampsia) and gestational diabetes mellitus (GDM) [6–8]. Furthermore, deliveries with a caesarean section have also increased, further complicating maternal-foetal outcomes [9], and leading to an increased prevalence of placenta previa and placenta accreta. Among these conditions, placenta accreta spectrum (PAS) is primarily the leading cause of severe postpartum haemorrhage (PPH), a condition characterised by severe cumulative blood loss of ≥500 mL within 24 hours post vaginal delivery or ≥1,000 mL after caesarean delivery. It also causes peripartum hysterectomy and even maternal mortality, thus, posing a major challenge in current obstetric clinical treatment [10,11]. Consequently, the correlation between fertility policy adjustments and obstetric health outcomes necessitates careful monitoring and thorough research.

While earlier research has provided preliminary insights into the impact of these policy adjustments on fertility, existing evidence still reveals clear literature gaps which makes it challenging to understand the effects of policy transitions fully. Many of the studies conducted have focused on comparing the late stages of the OCP and the early stages of the TCP in phases [5,12], overlooking the vital period following the implementation of the universal three-child policy (UCP) (2021–2025). As a result, these studies failed to fully capture the progression of impacts during the introduction and implementation of each policy. Second, the research time windows were relatively short, making it difficult to record the combined effects of changes in population baseline characteristics and the long-term cumulative repercussions of policy relaxation [13]. Lastly, even though maternal baseline characteristics (such as age, parity, and surgical history) are the fundamental premise for interpreting the variations in the risks of pregnancy complications, not enough attention has been given to the dynamic evolutionary trends of these characteristics across different policy periods. The previously mentioned knowledge gaps prevent both academics and policymakers from comprehensively assessing the real impacts of ongoing fertility policy relaxation on maternal and infant health. This limitation consequently restricts the effective distribution of perinatal healthcare resources and the precise formulation of management strategies for high-risk pregnant women.

This study is based on 15 years of consecutive electronic medical record data (2011–2025) obtained from a large regional medical centre in China that includes 43,439 deliveries. The study specifically addresses the OCP period (2011–2015), the TCP period (2016–2020), and the UCP period (2021–2025). The study aims to achieve three core objectives:

• to systematically describe and compare the dynamic changes in maternal demographic and obstetric baseline characteristics across the three policy periods, with a specific focus on the changes in the proportion of AMA mothers;

• to analyse the evolutionary patterns of pregnancy complication profiles, modes of delivery, and neonatal outcomes;

• to measure the effect of policy period as an independent variable on two key obstetric outcomes (caesarean section and PPH) while controlling for confounding maternal baseline factors, multivariate logistic regression was used.

By addressing the research gap in maternal and infant health studies throughout policy cycles, this research offers critical, evidence-based insights into the effects of scientifically evaluated fertility policies on public health. It also offers both theoretical significance and practical guiding value as it optimises the distribution of perinatal healthcare resources and establishes management systems for high-risk pregnant women.

## METHODS

### Research design and data sources

This study is a single-centre retrospective cohort study. All data were derived from the electronic medical record (EMR) system and the obstetric delivery registration database of a large tertiary grade A hospital in Guangxi Zhuang Autonomous Region, China. This database comprehensively recorded the admission information of pregnant women, prenatal examination materials, the entire process of delivery, and detailed data related to postpartum recovery. The study protocol was reviewed and approved by the hospital's institutional review committee (Approval Number: WM-2025 (137)); since this research involves a retrospective data analysis, the informed consent of patients was waived following the committee's approval.

### Research subjects and grouping

This study consecutively enrolled all parturients who delivered at our hospital between 1 January 2011 and 31 December 2025. Exclusion criteria were as follows:

• non-mainland Chinese parturients;

• cases with over 20% missing data on core variables, including delivery mode, birth weight, and gestational age;

• pregnancies terminated before 28 weeks of gestation.

The detailed participant screening process and the number of individuals excluded at each level are presented in Figure S1 in the **Online Supplementary Document**. Ultimately, a total of 43,439 eligible participants were included. Based on the timeline of the issuance and implementation of China's national family planning policies, all participants were divided into three cohorts: Group A (OCP, from 1 January 2011 to 31 December 31 2015, n = 13,268), Group B (TCP, from 1 January 2016 to 31 December 2020, n = 19,654), and Group C (UCP, from 1 January 2021 to 31 December 2025, n = 10,517).

### Research variables and definitions

Variables extracted from the database were divided into four categories: maternal baseline characteristics, pregnancy and delivery characteristics, intrapartum processes and outcomes, and other relevant variables. Maternal baseline characteristics included age (a continuous variable, further stratified into three groups: <35 years, 35≤age <40 years, and ≥40 years), gravidity, parity, marital status (married/unmarried), and ethnicity (Han/Zhuang/other minorities). Pregnancy and delivery characteristics included *in vitro* fertilisation (IVF) conception, twin pregnancy, premature rupture of membranes (PROM), HDP, GDM, placenta previa, placental abruption, foetal distress, and foetal growth restriction (FGR). Intrapartum processes and outcomes involved delivery mode (spontaneous vaginal delivery (SVD), instrumental vaginal delivery (forceps or vacuum extraction), and caesarean section; caesarean section classification was based on medical records), maternal outcomes (episiotomy, PPH), severe obstetric complications requiring intensive care unit (ICU) admission (with variable admission criteria across the study period), length of postpartum hospital stay, and neonatal outcomes (gestational age at delivery, birth weight, macrosomia (birth weight ≥4000 g), preterm birth (gestational age <37 weeks), and neonatal intensive care unit (NICU) admission). Other variables included admission route (outpatient/emergency/other) and ABO blood type.

Notably, the recorded rate of ICU admissions for obstetric patients showed a considerable decrease across study phases, which was attributed to the evolving institutional ICU admission policies rather than a reflection of the decreased disease severity. During the OCP period (2011–2015), the criteria for ICU admissions were relatively relaxed, allowing for a low threshold for high-risk deliveries. However, during the TCP period (2016–2020), a step-down unit was launched, which effectively reduced direct ICU transfers. In the UCP period (2021–2025), criteria became highly restrictive, with beds reserved exclusively for critically ill patients needing mechanical ventilation or vasopressors. Due to the absence of detailed administrative data, we could not perform relevant sensitivity analyses. Accordingly, cross-period comparisons of ICU admission rates should be interpreted with great caution.

### GDM diagnostic criteria and screening practices over time

The diagnostic criteria and screening protocols for GDM in our hospital were adjusted in accordance with the national guideline updates throughout the study period. From 2011 to 2015 (OCP period), GDM screening was based on risk factor assessment followed by a 50 g glucose challenge test (GCT). A 75 g oral glucose tolerance test (OGTT) was conducted solely for women who exhibited abnormal results from the GCT. In 2016–2020 (TCP period), China officially adopted the International Association of Diabetes and Pregnancy Study Groups (IADPSG) criteria, which advocate universal one-step 75 g OGTT at 24–28 weeks of gestation. However, universal OGTT screening was inconsistently implemented from 2016 to 2018 due to logistical constraints and delayed protocol adoption, and full implementation was achieved in 2019. Standard universal OGTT screening was consistently applied throughout 2021–2025 (UCP period).

### Statistical analysis

All statistical analyses were conducted using *R*, version 4.3.0 (R Foundation for Statistical Computing, Vienna, Austria). Normally distributed continuous variables were reported as mean ± standard deviation (SD) and compared across groups *via* one-way analysis of variance (ANOVA) with Tukey's honestly significant difference (HSD) post-hoc testing. Non-normally distributed continuous variables were presented as median (interquartile range, IQR) and analysed using the Kruskal-Wallis H test. Categorical variables were summarised as counts (n) and percentages, with between-group differences assessed using the χ^2^ test; Fisher exact test was adopted when the expected cell frequencies were below 5.

Multivariate logistic regression models were applied to evaluate the independent association between policy period and primary outcomes (caesarean section and PPH), with the OCP period set as the reference group. Covariates were selected using a two-step procedure. First, all candidate variables were examined by univariate logistic regression, and those with *P* < 0.10 were kept to avoid losing any potential confounders. Second, variables were divided into confounders and mediators according to causal pathways. Mediators, including maternal age, parity, AMA status, nulliparity, previous uterine scar, and HDP, as well as GDM (excluded due to screening-related recording bias), were all removed to avoid overadjustment bias. The final covariates were twin pregnancy, placenta previa, macrosomia, and preterm birth; delivery mode was further adjusted in the PPH model. Episiotomy and instrumental delivery were excluded from primary analyses because of incomplete documentation during the TCP period. All tests were two-tailed, and a *P*-value <0.05 indicated statistical significance. The reported adjusted odds ratios (aORs) reflect the direct effects of the policy period.

## RESULTS

### Baseline characteristics of study subjects

A total of 43,439 deliveries were included in the final analysis. Significant changes were observed in the demographic and obstetric characteristics of pregnant women during different policy periods (**Table 1**). The average maternal age increased consistently, rising from 27.58 years in the OCP period to 29.96 years in the TCP period, and reaching 31.37 years in the UCP period (*P* < 0.001). The proportion of AMA women also increased substantially from 9.3% in the OCP period to 30.5% in the UCP period (*P* < 0.001), with the proportion of pregnant women aged ≥40 years rising from 1.4% to 6.7% (*P* < 0.001).

**Table 1:** Demographic and obstetric characteristics across three policy periods

Variables	OCP (2011–2015) (n = 13,268)	TCP (2016–2020) (n = 19,654)	UCP (2021–2025) (n = 10,517)	F/χ^2^	*P-*value
Maternal age	27.58 ± 4.85	29.96 ± 5.36	31.37 ± 5.57	F = 1616.00	<0.001
Neonatal birth weight	3,133.35 ± 406.21	3,164.74 ± 448.65	3,156.91 ± 458.72	F = 11.65	<0.001
Actual hospitalisation	5.82 ± 3.10	5.18 ± 2.27	4.73 ± 2.15	F = 569.85	<0.001
Gestational age	38.04 ± 1.76	38.45 ± 1.51	38.54 ± 1.42	F = 364.61	<0.001
Gravidity	1.78 ± 0.90	3.00 ± 0.82	2.75 ± 1.50	F = 1919.14	<0.001
Parity	0.39 ± 0.59	0.74 ± 0.67	0.86 ± 0.79	F = 1635.48	<0.001
Marital status				χ^2^ = 56.15	<0.001
*Unmarried*	689 (5.2%)	1,161 (5.9%)	788 (7.5%)		
*Married*	12,579 (94.8%)	18,493 (94.1%)	9,728 (92.5%)		
Ethnicity				χ^2^ = 317.68	<0.001
*Han*	1,398 (10.5%)	3,352 (17.1%)	1,496 (14.2%)		
*Minority*	199 (1.5%)	446 (2.3%)	160 (1.5%)		
*Zhuang*	11,664 (87.9%)	15,853 (80.7%)	8,840 (84.0%)		
AMA				χ^2^ = 1710.20	<0.001
*Yes*	1,239 (9.3%)	3,848 (19.6%)	3,211 (30.5%)		
*No*	12,028 (90.7%)	15,806 (80.4%)	7,306 (69.5%)		
Age >40 years				χ^2^ = 446.15	<0.001
*Yes*	189 (1.4%)	788 (4.0%)	709 (6.7%)		
*No*	13,079 (98.6%)	18,866 (96.0%)	9,808 (93.3%)		
Nulliparous				χ^2^ = 2796.79	<0.001
*Yes*	8,799 (66.3%)	7,522 (38.3%)	4,200 (39.9%)		
*No*	4,469 (33.7%)	12,132 (61.7%)	6,317 (60.1%)		
Twin pregnancy				χ^2^ = 0.70	0.706
*Yes*	188 (1.4%)	296 (1.5%)	162 (1.5%)		
*No*	13,080 (98.6%)	19,358 (98.5%)	10,355 (98.5%)		
IVF conception				χ^2^ = 534.68	<0.001
*Yes*	174 (1.3%)	466 (2.4%)	654 (6.2%)		
*No*	13,094 (98.7%)	19,188 (97.6%)	9,863 (93.8%)		
Premature rupture of membranes				χ^2^ = 545.66	<0.001
*Yes*	1,448 (10.9%)	3,465 (17.6%)	2,314 (22.0%)		
*No*	11,820 (89.1%)	16,189 (82.4%)	8,203 (78.0%)		
HDP				χ^2^ = 395.05	<0.001
*Yes*	563 (4.2%)	405 (2.1%)	696 (6.6%)		
*No*	12,705 (95.8%)	19,249 (97.9%)	9,821 (93.4%)		
GDM				χ^2^ = 4168.08	<0.001
*Yes*	1,357 (10.2%)	506 (2.6%)	2,811 (26.7%)		
*No*	11,911 (89.8%)	19,148 (97.4%)	7,706 (73.3%)		
Placenta previa				χ^2^ = 35.22	<0.001
*Yes*	112 (0.8%)	312 (1.6%)	154 (1.5%)		
*No*	13,156 (99.2%)	19,342 (98.4%)	10,363 (98.5%)		
Placental abruption				χ^2^ = 156.15	<0.001
*Yes*	35 (0.3%)	115 (0.6%)	169 (1.6%)		
*No*	13,233 (99.7%)	19,539 (99.4%)	10,348 (98.4%)		
Foetal distress				χ^2^ = 74.40	<0.001
*Yes*	848 (6.4%)	861 (4.4%)	476 (4.5%)		
*No*	12,420 (93.6%)	18,793 (95.6%)	10,041 (95.5%)		
FGR				χ^2^ = 8.16	0.017
*Yes*	159 (1.2%)	289 (1.5%)	171 (1.6%)		
*No*	13,109 (98.8%)	19,365 (98.5%)	10,346 (98.4%)		
Caesarean delivery				χ^2^ = 290.19	<0.001
*Yes*	3,926 (29.6%)	6,445 (32.8%)	4,199 (39.9%)		
*No*	9,342 (70.4%)	13,209 (67.2%)	6,318 (60.1%)		
Episiotomy				χ^2^ = NaN	-
*Yes*	1,374 (10.4%)	0 (0.0%)	703 (6.7%)		
*No*	11,894 (89.6%)	0 (0.0%)	9,621 (91.5%)		
Instrumental delivery				χ^2^ = NaN	-
*Yes*	8 (0.1%)	0 (0.0%)	61 (0.6%)		
*No*	13,260 (99.9%)	0 (0.0%)	10,263 (97.6%)		
Uterine scar				χ^2^ = 888.79	<0.001
*Yes*	1,005 (7.6%)	3,430 (17.4%)	2,131 (20.3%)		
*No*	12,263 (92.4%)	16,224 (82.5%)	8,386 (79.7%)		
PPH				χ^2^ = 110.25	<0.001
*Yes*	253 (1.9%)	766 (3.9%)	291 (2.8%)		
*No*	13,015 (98.1%)	18,888 (96.1%)	10,226 (97.2%)		
Macrosomia				χ^2^ = 146.79	<0.001
*Yes*	153 (1.1%)	632 (3.2%)	307 (2.9%)		
*No*	13,115 (98.8%)	19,022 (96.8%)	10,210 (97.1%)		
Preterm birth				χ^2^ = 284.99	<0.001
*Yes*	317 (2.4%)	1,244 (6.3%)	638 (6.1%)		
*No*	12,951 (97.6%)	18,410 (93.7%)	9,879 (93.9%)		
Critical illness				χ^2^ = 1707.96	<0.001
*Yes*	1899 (14.3%)	947 (4.8%)	173 (1.6%)		
*No*	11,369 (85.7%)	18,707 (95.2%)	10,344 (98.4%)		
Admission route				χ^2^ = 3451.06	<0.001
*Emergency*	1,574 (11.9%)	6,698 (34.1%)	4,764 (45.3%)		
*Outpatient*	11,655 (87.8%)	12,852 (65.4%)	5,747 (54.6%)		
*Other*	39 (0.3%)	104 (0.5%)	6 (0.1%)		
Blood type				χ^2^ = 1274.59	<0.001
*Type AB*	2,007 (15.1%)	1,007 (5.1%)	570 (5.4%)		
*Type A*	2,864 (21.6%)	4,186 (21.3%)	2,239 (21.3%)		
*Type B*	3,621 (27.3%)	5,569 (28.3%)	2,822 (26.8%)		
*Type O*	4,776 (36.0%)	8,892 (45.2%)	4,694 (44.6%)		

AMA – advanced maternal age, FGR – foetal growth restriction, GDM – gestational diabetes mellitus, HDP – hypertensive disorders of pregnancy, IVF – *in vitro* fertilisation, OCP – one-child policy, PPH – postpartum haemorrhage, TCP – two-child policy, UCP – universal three-child policy

Notable changes were identified in reproductive history. The proportion of primiparas decreased significantly from 66.3% in the OCP period to 38.3% in the TCP period, and remained relatively stable at 39.9% in the UCP period (*P* < 0.001). Corresponding increases were observed in the average gravidity and parity (both *P* < 0.001). The utilisation rate of IVF increased markedly over time, rising from 1.3% in the OCP period to 6.2% in the UCP period (*P* < 0.001), which reflects the age-related decline in fertility and the consequent increase in medical interventions [14].

Other characteristics, including a slight increase in the proportion of unmarried women, a slight decrease in the proportion of Zhuang ethnicity (the predominant ethnic group), and changes in blood type distribution, all showed statistically significant differences (all *P* < 0.001). The only characteristic that remained stable was the incidence of twin pregnancy, which ranged from 1.4% to 1.5% across the three periods with no significant difference (*P* = 0.706).

### Trends in pregnancy complications and delivery-related indicators

The incidence of pregnancy complications across different policy periods showed significant changes (**Table 1**). The prevalence of GDM showed fluctuations, decreasing to 2.6% during the TCP period and then sharply increasing to 26.7% in the UCP period (*P* < 0.001). This U-shaped pattern is largely driven by successive updates to national GDM diagnostic criteria and institutional screening protocols, rather than true epidemiological shifts. During the TCP period, China implemented the IADPSG criteria, which advocate for a universal one-step 75 g OGTT for all pregnant women between 24 and 28 weeks of gestation. However, from 2016 to 2018, the implementation of the universal OGTT screening was inconsistent due to logistical challenges, which resulted in notable underdiagnoses. The protocol was fully implemented from 2019 onwards and maintained throughout the UCP period. Consequently, the recorded GDM prevalence should not be interpreted as reflecting true disease burden. Detailed information on screening practices, diagnostic criteria and OGTT coverage rates across periods is provided (Table S1 in the **Online Supplementary Document**). The incidences of placental abruption and placenta previa both increased progressively with the advancement of policy periods (both *P* < 0.001). The incidence of premature rupture of membranes (PROM) increased from 10.9% in the OCP period to 22.0% in the UCP period (*P* < 0.001). The incidence of HDP was lowest in the TCP period (2.1%). However, it was relatively higher in the OCP and UCP periods, with rates of 4.2% and 6.6%, respectively, showing a statistically significant overall difference (*P* < 0.001).

Regarding delivery-related indicators, the average gestational age at delivery slightly increased from 38.04 weeks in the OCP period to 38.54 weeks in the UCP period (*P* < 0.001). The average birth weight of neonates showed mild fluctuations across the three periods, but there was a significant difference (*P* < 0.001), with the mean values ranging from 3,156 g to 3,165 g. The incidence of macrosomia rose from 1.1% in the OCP period to 2.9% in the UCP period (*P* < 0.001), while the incidence of FGR experienced a slight increase, from 1.2% to 1.6% (*P* = 0.017). The preterm birth rate significantly increased from 2.4% in the OCP period to 6.3% in the TCP period and 6.1% in the UCP period (*P* < 0.001). For episiotomy and instrumental delivery, zero cases were recorded during the TCP period (2016–2020) due to inconsistent documentation in electronic medical records. These zero values do not represent the actual incidence of the two indicators.

### Changes in delivery modes and maternal outcomes

The overall caesarean section rate increased steadily, increasing from 29.6% in the OCP period to 32.8% in the TCP period, and further rising to 39.9% in the UCP period (*P* < 0.001). The incidence of PPH peaked at 3.9% in the TCP period and then decreased to 2.8% in the UCP period. Despite this reduction, the rate was still higher than in the OCP period (1.9%), highlighting a significant overall difference (*P* < 0.001). The utilisation rate of intrapartum analgesia increased remarkably, from 7.6% in the OCP period to 20.3% in the UCP period (*P* < 0.001).

A notable change was seen in the admission route of pregnant women. The proportion of pregnant women admitted to the emergency department rose sharply from 11.9% in the OCP period to 45.3% in the UCP period (*P* < 0.001). In contrast, the average length of hospital stay was significantly shortened, decreasing from 5.82 days in the OCP period to 4.73 days in the UCP period (*P* < 0.001). The incidence of severe pregnancy-related complications was relatively higher during the OCP period at 14.3%, which subsequently decreased to 4.8% in the TCP period and 1.6% in the UCP period (*P* < 0.001). Rather than a true decrease in clinical severity, this significant dip is probably the consequence of changes in ICU admission criteria and documentation practice.

### Subgroup analysis of AMA and non-AMA

Subgroup analyses were performed to explore differences in pregnancy outcomes between AMA and non-AMA parturients (**Table 2**, **Table 3**). Logistic regression models with a policy period-AMA status interaction term were used to test effect modification, adjusting for twin pregnancy, placenta previa, macrosomia, and preterm birth; delivery mode was further controlled in analyses of PPH. Interaction tests showed that there was no notable effect change based on AMA status in relation to caesarean section (TCP *vs.* OCP: *P* = 0.18; UCP *vs.* OCP: *P* = 0.24) and PPH (TCP *vs.* OCP: *P* = 0.32; UCP *vs.* OCP: *P* = 0.41) (Table S2 in the **Online Supplementary Document**). The associations between policy period and the two pregnancy outcomes were consistent across AMA and non-AMA groups. Thus, subgroup data are presented descriptively without formal statistical comparisons between subgroups. Additionally, it is important to cautiously interpret GDM prevalence as there have been variations in screening coverage over time (Table S1 in the **Online Supplementary Document**).

**Table 2:** Demographic and obstetric characteristics of AMA women across three policy periods

Variables	OCP (2011–2015) (n = 12,028)	TCP (2016–2020) (n = 15,806)	UCP (2021–2025) (n = 7,306)	F/χ^2^	*P-*value
Maternal age	37.24 ± 2.14	37.64 ± 2.42	37.73 ± 2.51	F = 19.46	<0.001
Neonatal birth weight	3,141.68 ± 426.21	3,165.58 ± 480.36	3,160.19 ± 484.67	F = 0.72	0.486
Actual hospitalisation	6.43 ± 3.54	5.52 ± 2.51	4.82 ± 2.19	F = 185.93	<0.001
Gestational age	37.92 ± 1.76	38.29 ± 1.56	38.37 ± 1.47	F = 36.90	<0.001
Gravidity	2.18 ± 0.95	3.00 ± 0.00	2.96 ± 1.58	F = 133.54	<0.001
Parity	0.80 ± 0.68	1.06 ± 0.61	0.98 ± 0.82	F = 60.67	<0.001
Marital status				χ^2^ = 02.21	<0.001
*Unmarried*	13 (1.0%)	66 (1.7%)	175 (5.4%)		
*Married*	1,226 (99.0%)	3,782 (98.3%)	3,035 (94.5%)		
Ethnicity				χ^2^ = 6.80	0.147
*Han*	130 (10.5%)	482 (12.5%)	369 (11.5%)		
*Minority*	16 (1.3%)	73 (1.9%)	52 (1.6%)		
*Zhuang*	1,093 (88.2%)	3,293 (85.6%)	2,786 (86.8%)		
Advanced maternal age				χ^2^ = 25.84	<0.001
*35–40 years*	1,050 (84.8%)	3,060 (79.5%)	2,502 (77.9%)		
*>40 years*	189 (15.2%)	788 (20.5%)	709 (22.1%)		
Age >40 years					
*Yes*	189 (15.2%)	788 (20.5%)	709 (22.1%)	χ^2^ = 25.84	<0.001
*No*	1,050 (84.8%)	3,060 (79.5%)	2,502 (77.9%)		
Nulliparous				χ^2^ = 381.48	<0.001
*Yes*	432 (34.9%)	537 (14.0%)	994 (31.0%)		
*No*	807 (65.1%)	3,311 (86.0%)	2,217 (69.0%)		
Twin pregnancy				χ^2^ = 1.07	0.587
*Yes*	24 (1.9%)	62 (1.6%)	61 (1.9%)		
*No*	1,215 (98.1%)	3,786 (98.4%)	3,150 (98.1%)		
IVF conception				χ^2^ = 114.68	<0.001
*Yes*	60 (4.8%)	175 (4.5%)	346 (10.8%)		
*No*	1,179 (95.2%)	3,673 (95.5%)	2,865 (89.2%)		
Premature rupture of membranes				χ^2^ = 44.63	<0.001
*Yes*	159 (12.8%)	687 (17.9%)	685 (21.3%)		
*No*	1,080 (87.2%)	3,161 (82.2%)	2,526 (78.7%)		
HDP				χ^2^ = 80.15	<0.001
*Yes*	95 (7.7%)	134 (3.5%)	267 (8.3%)		
*No*	1,144 (92.3%)	3,714 (96.5%)	2,944 (91.7%)		
GDM				χ^2^ = 1327.03	<0.001
*Yes*	250 (20.2%)	144 (3.7%)	1,229 (38.3%)		
*No*	989 (79.8%)	3,704 (96.3%)	1,982 (61.7%)		
Placenta previa				χ^2^ = 7.08	0.029
*Yes*	22 (1.8%)	124 (3.2%)	91 (2.8%)		
*No*	1,217 (98.2%)	3,724 (96.8%)	3,120 (97.2%)		
Placental abruption				χ^2^ = 13.35	0.001
*Yes*	10 (0.8%)	38 (1.0%)	60 (1.9%)		
*No*	1,229 (99.2%)	3,810 (99.0%)	3,151 (98.1%)		
Foetal distress				χ^2^ = 5.64	0.060
*Yes*	74 (6.0%)	192 (5.0%)	138 (4.3%)		
*No*	1,165 (94.0%)	3,656 (95.0%)	3,073 (95.4%)		
FGR				χ^2^ = 3.70	0.157
*Yes*	13 (1.0%)	64 (1.7%)	60 (1.9%)		
*No*	1,226 (99.0%)	3,784 (98.3%)	3,151 (98.1%)		
Caesarean delivery				χ^2^ = 46.38	<0.001
*Yes*	561 (45.3%)	1,773 (46.1%)	1,719 (53.5%)		
*No*	678 (54.7%)	2,075 (53.9%)	1,492 (46.5%)		
Episiotomy				χ^2^ = NaN	-
*Yes*	48 (3.9%)	0 (0.0%)	107 (3.3%)		
*No*	1,191 (96.1%)	0 (0.0%)	3,042 (94.7%)		
Instrumental delivery				χ^2^ = NaN	-
*Yes*	0 (0.0%)	0 (0.0%)	9 (0.3%)		
*No*	1,239 (100.0%)	0 (0.0%)	3,140 (97.8%)		
Maternal pain management				χ^2^ = 120.95	<0.001
*Yes*	194 (15.7%)	1,046 (27.2%)	1,029 (32.0%)		
*No*	1,045 (84.3%)	2,802 (72.8%)	2,182 (68.0%)		
PPH				χ^2^ = 9.42	0.009
*Yes*	33 (2.7%)	176 (4.6%)	122 (3.8%)		
*No*	1,206 (97.3%)	3,672 (95.4%)	3,089 (96.2%)		
Macrosomia				χ^2^ = 11.50	0.003
*Yes*	22 (1.8%)	144 (3.7%)	112 (3.5%)		
*No*	1,217 (98.2%)	3,704 (96.3%)	3,099 (96.5%)		
Preterm birth				χ^2^ = 30.01	<0.001
*Yes*	45 (3.6%)	317 (8.2%)	243 (7.6%)		
*No*	1,194 (96.4%)	3,531 (91.8%)	2,968 (92.4%)		
Critical illness				χ^2^ = 164.12	<0.001
*Yes*	157 (12.7%)	266 (6.9%)	85 (2.6%)		
*No*	1,082 (87.3%)	3,582 (93.1%)	3,126 (97.3%)		
Admission route				χ^2^ = 378.88	<0.001
*Emergency*	131 (10.6%)	1,291 (33.5%)	1,316 (41.0%)		
*Outpatient*	1,102 (88.9%)	2,541 (66.0%)	1,891 (58.9%)		
*Other*	6 (0.5%)	16 (0.4%)	4 (0.1%)		
Blood type				χ^2^ = 146.04	<0.001
*Type AB*	182 (14.7%)	202 (5.2%)	176 (5.5%)		
*Type A*	257 (20.7%)	821 (21.3%)	688 (21.4%)		
*Type B*	326 (26.3%)	1,112 (28.9%)	884 (27.5%)		
*Type O*	474 (38.3%)	1,713 (44.5%)	1,401 (43.6%)		

AMA – advanced maternal age, FGR – foetal growth restriction, GDM – gestational diabetes mellitus, HDP – hypertensive disorders of pregnancy, IVF – *in vitro* fertilisation, OCP – one-child policy, PPH – postpartum haemorrhage, TCP – two-child policy, UCP – universal three-child policy.

**Table 3:** Demographic and obstetric characteristics of non-AMA women across three policy periods

Variables	OCP (2011–2015) (n = 12,028)	TCP (2016–2020) (n = 15,806)	UCP (2021–2025) (n = 7,306)	F/χ^2^	*P-*value
Maternal age	26.59 ± 3.86	28.09 ± 4.05	28.58 ± 4.05	F = 719.40	<0.001
Neonatal birth weight	3,132.34 ± 403.76	3,164.54 ± 440.60	3,155.47 ± 446.87	F = 11.12	<0.001
Actual hospitalisation	5.76 ± 3.04	5.09 ± 2.20	4.70 ± 2.13	F = 458.17	<0.001
Gestational age	38.06 ± 1.76	38.48 ± 1.50	38.61 ± 1.39	F = 369.97	<0.001
Gravidity	1.74 ± 0.89	3.00 ± 1.00	2.66 ± 1.45	F = 1503.16	<0.001
Parity	0.35 ± 0.56	0.66 ± 0.67	0.81 ± 0.77	F = 1320.30	<0.001
Marital status				χ^2^ = 56.09	<0.001
*Unmarried*	676 (5.6%)	1,095 (6.9%)	613 (8.4%)		
*Married*	11,352 (94.4%)	14,711 (93.1%)	6,693 (91.6%)		
Ethnicity				χ^2^ = 357.41	<0.001
*Han*	1,268 (10.5%)	2,870 (18.2%)	1,127 (15.4%)		
*Minority*	183 (1.5%)	373 (2.4%)	108 (1.5%)		
*Zhuang*	10,570 (87.9%)	12,560 (79.5%)	6,054 (82.9%)		
AMA				χ^2^ = NaN	-
*Yes*	0 (0.0%)	0 (0.0%)	0 (0.0%)		
*No*	12,028 (100.0%)	15,806 (100.0%)	7,306 (100.0%)		
Nulliparous				χ^2^ = 2057.94	<0.001
*Yes*	8,366 (69.5%)	6,985 (44.2%)	3,206 (43.9%)		
*No*	3,662 (30.4%)	8,821 (55.8%)	4,100 (56.1%)		
Twin pregnancy				χ^2^ = 0.76	0.684
*Yes*	164 (1.4%)	234 (1.5%)	101 (1.4%)		
*No*	11,864 (98.6%)	15,572 (98.5%)	7,205 (98.6%)		
IVF conception				χ^2^ = 249.29	<0.001
*Yes*	114 (0.9%)	291 (1.8%)	308 (4.2%)		
*No*	11,914 (99.0%)	15,515 (98.2%)	6,998 (95.8%)		
Premature rupture of membranes				χ^2^ = 489.11	<0.001
*Yes*	1,288 (10.7%)	2,778 (17.6%)	1,629 (22.3%)		
*No*	10,740 (89.3%)	13,028 (82.4%)	5,677 (77.7%)		
HDP				χ^2^ = 287.04	<0.001
*Yes*	468 (3.9%)	271 (1.7%)	429 (5.9%)		
*No*	11,560 (96.1%)	15,535 (98.3%)	6,877 (94.1%)		
GDM				χ^2^ = 2369.04	<0.001
*Yes*	1,107 (9.2%)	362 (2.3%)	1,582 (21.6%)		
*No*	10,921 (90.8%)	15,444 (97.7%)	5,724 (78.3%)		
Placenta previa				χ^2^ = 14.96	<0.001
*Yes*	90 (0.8%)	188 (1.2%)	63 (0.9%)		
*No*	11,938 (99.2%)	15,618 (98.8%)	7,243 (99.1%)		
Placental abruption				χ^2^ = 131.74	<0.001
*Yes*	25 (0.2%)	77 (0.5%)	109 (1.5%)		
*No*	12,003 (99.8%)	15,729 (99.5%)	7,197 (98.5%)		
Foetal distress				χ^2^ = 72.60	<0.001
*Yes*	774 (6.4%)	669 (4.2%)	338 (4.6%)		
*No*	11,254 (93.6%)	15,137 (95.8%)	6,968 (95.4%)		
FGR				χ^2^ = 3.71	0.157
*Yes*	146 (1.2%)	225 (1.4%)	111 (1.5%)		
*No*	11,882 (98.8%)	15,581 (98.6%)	7,195 (98.5%)		
Caesarean delivery				χ^2^ = 79.08	<0.001
*Yes*	3,365 (28.0%)	4,672 (29.6%)	2,480 (33.9%)		
*No*	8663 (72.0%)	11,134 (70.4%)	4,826 (66.1%)		
Episiotomy				χ^2^ = NaN	-
*Yes*	1,326 (11.0%)	0 (0.0%)	596 (8.2%)		
*No*	10,702 (89.0%)	0 (0.0%)	6,579 (90.0%)		
Instrumental delivery				χ^2^ = NaN	-
*Yes*	8 (0.1%)	0 (0.0%)	52 (0.7%)		
*No*	12,020 (99.9%)	0 (0.0%)	7,123 (97.5%)		
Uterine scar				χ^2^ = 512.73	<0.001
*Yes*	811 (6.7%)	2,384 (15.1%)	1,102 (15.1%)		
*No*	11,217 (93.3%)	13,422 (84.9%)	6,204 (84.9%)		
PPH				χ^2^ = 99.01	<0.001
*Yes*	220 (1.8%)	590 (3.7%)	169 (2.3%)		
*No*	11,808 (98.2%)	15,216 (96.3%)	7,137 (97.7%)		
Macrosomia				χ^2^ = 125.60	<0.001
*Yes*	131 (1.1%)	488 (3.1%)	195 (2.7%)		
*No*	11,897 (98.9%)	15,318 (96.9%)	7,111 (97.3%)		
Preterm birth				χ^2^ = 220.95	<0.001
*Yes*	272 (2.3%)	927 (5.9%)	395 (5.4%)		
*No*	11,756 (97.7%)	14,879 (94.1%)	6,911 (94.6%)		
Critical illness				χ^2^ = 1556.33	<0.001
*Yes*	1,742 (14.5%)	681 (4.3%)	88 (1.2%)		
*No*	10,286 (85.5%)	15,125 (95.7%)	7,218 (98.8%)		
Admission route				χ^2^ = 3097.75	<0.001
*Emergency*	1,442 (12.0%)	5,407 (34.2%)	3,448 (47.2%)		
*Outpatient*	10,553 (87.7%)	10,311 (65.2%)	3,856 (52.8%)		
*Other*	33 (0.3%)	88 (0.6%)	2 (0.0%)		
Blood type				χ^2^ = 1089.28	<0.001
*Type AB*	1,824 (15.2%)	805 (5.1%)	394 (5.4%)		
*Type A*	2,607 (21.7%)	3,365 (21.3%)	1,551 (21.2%)		
*Type B*	3,295 (27.4%)	4,457 (28.2%)	1,938 (26.5%)		
*Type O*	4,302 (35.8%)	7,179 (45.4%)	3,293 (45.1%)		

AMA – advanced maternal age, FGR – foetal growth restriction, GDM – gestational diabetes mellitus, HDP – hypertensive disorders of pregnancy, IVF – *in vitro* fertilisation, OCP – one-child policy, PPH – postpartum haemorrhage, TCP – two-child policy, UCP – universal three-child policy

For completeness, stratified subgroup data are presented descriptively. The mean maternal age in the AMA group rose slightly from 37.24 years during the OCP period to 37.73 years during the UCP period, with a statistically significant difference (*P* < 0.001) (**Table 2**). The prevalence of GDM in this group varied markedly, peaking at 38.3% in the UCP period (Table S1 in the **Online Supplementary Document**). The caesarean section rate rose from 45.3% in the OCP period to 53.5% in the UCP period. Notably, the proportion of patients with a uterine scar increased significantly from 15.7% to 32.0% (*P* < 0.001), accompanied by a substantial surge in the rate of emergency hospital admission from 10.6% to 41.0%. In the non-AMA group (**Table 3**), the mean maternal age increased from 26.59 years in the OCP period to 28.58 years in the UCP period (*P* < 0.001). The prevalence of GDM showed a U-shaped trend across the periods, with values of 9.2% (OCP), 2.3% (TCP), and 21.6% (UCP), respectively (Table S1 in the **Online Supplementary Document**). The caesarean section rate increased from 28.0% to 33.9% in this group. Most notably, the rate of emergency hospitalisation increased from 12.0% to 47.2%, and the rate of premature birth increased from 2.3% to 5.4%.

### Multivariate logistic regression analysis

#### Factors related to caesarean section

Once multiple confounders were accounted for, the policy period was identified as an independent variable associated with caesarean deliveries. Compared with the OCP period, the risk of caesarean section decreased by 9% during the TCP period (aOR = 0.91; 95% confidence interval (CI) = 0.86–0.95), while it rose by 8% in the UCP period (aOR = 1.08; 95% CI = 1.02–1.15) (overall *P* < 0.001). These aORs only show the direct effects of the policy period, excluding mediating effects of maternal age, parity, and prior uterine scar. Crude odds ratios reflecting the total effects are listed (Table S3 in the **Online Supplementary Document**). To avoid overadjustment bias, maternal age, parity, and other mediators were not incorporated into the primary model as covariates, and their independent adjusted effects were therefore not presented. Additionally, Twin pregnancy (aOR = 11.90; 95% CI = 9.53–15.10, *P* < 0.001) and placenta previa (aOR = 12.80; 95% CI = 9.96–16.70, *P* < 0.001) were the leading risk factors for caesarean section, while macrosomia (aOR = 2.23; 95% CI = 2.05–2.63, *P* < 0.001) and preterm birth (aOR = 1.51; 95% CI = 1.35–1.69, *P* < 0.001) also significantly elevated caesarean delivery risk.

#### Factors related to PPH

Policy periods were significantly associated with PPH. The TCP period had the highest PPH risk (aOR = 1.80; 95% CI = 1.55–2.10), and the UCP period also showed a higher PPH risk relative to the OCP period (aOR = 1.24; 95% CI = 1.03–1.49) (overall *P* < 0.001). Placenta previa was identified as the predominant risk factor for PPH (aOR = 13.70; 95% CI = 10.50–17.70, *P* < 0.001), followed by foetal macrosomia (aOR = 3.08; 95% CI = 2.41–3.89, *P* < 0.001) and preterm birth (aOR = 2.08; 95% CI = 1.57–2.74, *P* < 0.001). Multivariate regression analysis indicated that caesarean delivery was correlated with a lower incidence of PPH (aOR = 0.28; 95% CI = 0.24–0.33, *P* < 0.001). This association does not indicate a true protective effect of caesarean section against PPH, as the diagnostic thresholds differ by delivery mode: PPH is defined as blood loss ≥500 mL for vaginal delivery and ≥1000 mL for caesarean section. This discrepancy leads to measurement bias and artificially lowers the PPH detection rate in caesarean deliveries, rendering cross-group comparisons invalid. Therefore, this result is not considered clinically meaningful and has been removed from the core findings of the present study.

### Visualisation of time trend

**Figure 1:**
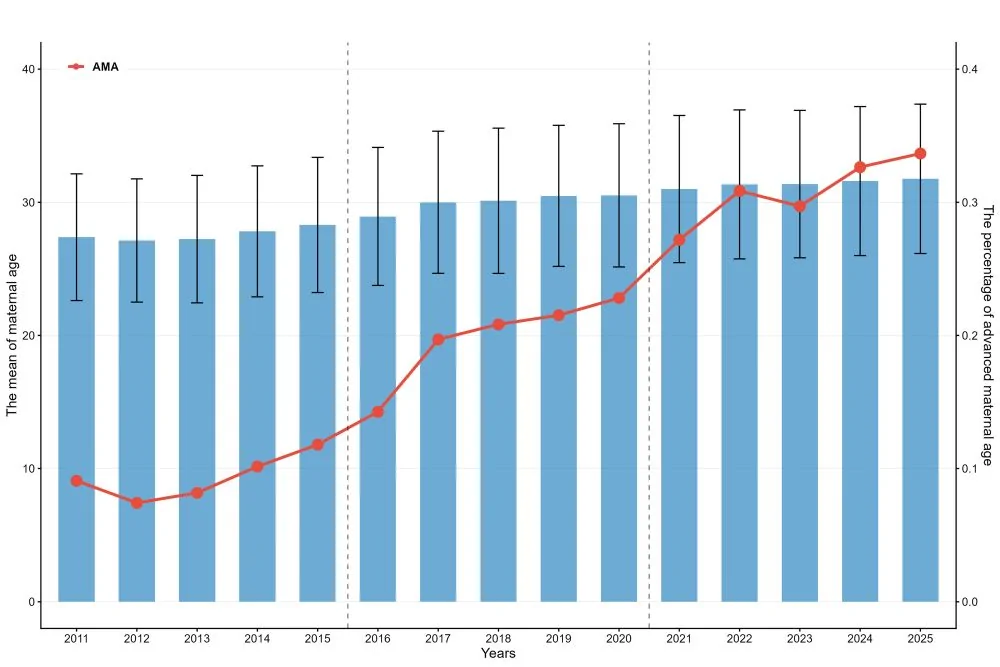
Trends in maternal age and AMA from 2011 to 2025. AMA – advanced maternal age.

**Figure 2:**
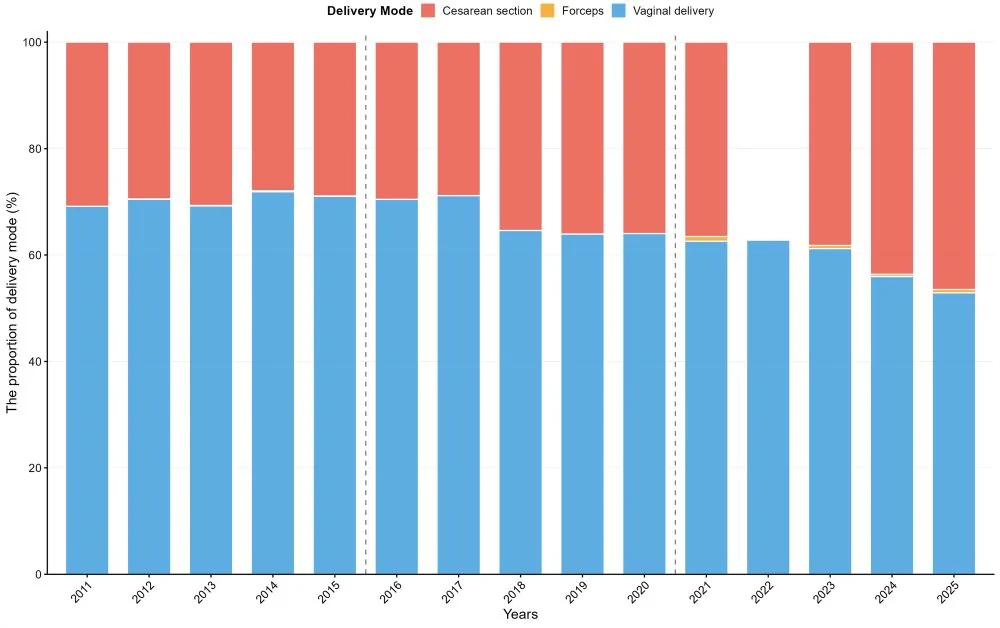
Proportions of delivery modes from 2011 to 2025.

**Figure 3:**
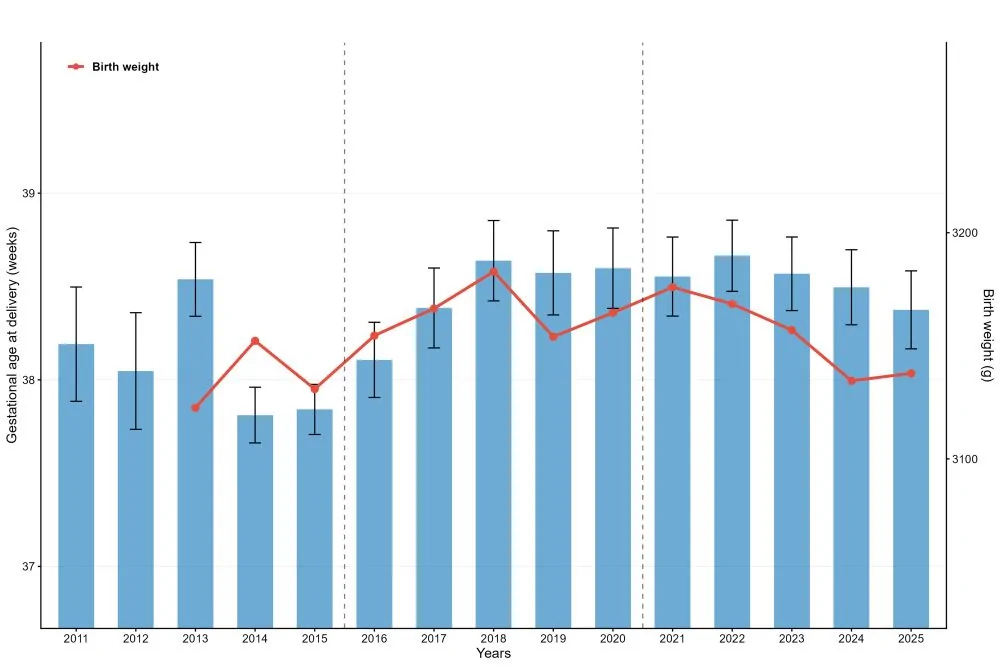
Trends in gestational age and birth weight from 2011 to 2025.

The steady increase in the average maternal age (from approximately 28 to 32 years) and the significant rise in the percentage of AMA women (from around 9% to over 33%) during 2011–2025 were illustrated (**Figure 1**). The stacked bar chart revealed a remarkable shift in the age distribution of parturients: the percentage of parturients aged <35 years steadily declined, while the percentages of those aged 35–39 years and ≥40 years old steadily increased (**Figure 2**). The gestational age at delivery stabilised at a relatively high level in the later period (approximately 38.5 weeks), while the birth weight peaked in the middle period, then declined slightly and stabilised (**Figure 3**).

## DISCUSSION

This study employed 15-year cohort data from a tertiary hospital encompassing over 43,000 deliveries across three consecutive Chinese fertility policy eras to explore associations between fertility policy reforms and changes in maternal demographic characteristics, gestational complication spectrum, delivery modes, and obstetric healthcare utilisation. The core findings showed a time-dependent correlation between policy changes and the emergence of a maternal triple-high phenotype (AMA, high parity, increased ART conception), which functioned as the primary biological driver for increased obstetric risks during the study period. Notably, policy periods were confounded by secular updates of clinical practices, diagnostic criteria, healthcare service delivery, and patients' medical-seeking behaviours. As a result, all observed correlations were temporal associations and did not signify a causal relationship.

### Altered maternal population structure: triple-high trend and clinical implications

Consistent with previous domestic epidemiological studies [7,15], a 4-year rise in mean maternal age and a more than 3-fold increase in AMA proportion were observed in this cohort from the OCP period to the UCP period, with a larger growth amplitude than the nationally published data. Existing clinical evidence has validated that AMA independently elevates risks of GDM, HDP, preterm birth, and foetal chromosomal abnormalities [16]. Decreased primipara ratio, expanded multiparous population, and growing scar uterus prevalence collectively increased the incidence of placenta previa and PAS disorders [10,17]. The proportion of doubled scarring on the uterus among AMA women further confirms that prior caesarean delivery is the dominant risk factor for PAS [10]. Furthermore, rising ART utilisation driven by age-related fertility decline stabilised the overall twin pregnancy rate, while increasing subsequent risks of preterm birth and caesarean delivery [18,19].

### Evolution of pregnancy complication spectrum: GDM surge and gestational metabolic management

During the UCP period, the overall prevalence of GDM was 26.7%, peaking at 38.3% in the AMA subgroup. This prevalence was higher than that of other global cohorts but aligned with recent population-based surveys conducted in China [20,21]. The U-shaped trend of recorded GDM prevalence (10.2%→2.6%→26.7%) was primarily linked to the gradual implementation of IADPSG criteria and inconsistent GDM screening protocols during the TCP period, rather than true epidemiological fluctuations. Universal OGTT screening was not fully implemented in this hospital until the late TCP period (2019), resulting in underdiagnosis and artificially low GDM prevalence (2.6%) during 2016–2020. By contrast, sustained universal OGTT screening in the UCP period reflected the actual GDM disease burden of the study population. The rising GDM prevalence was also linked to AMA accumulation, altered dietary and lifestyle patterns among reproductive-age females, and a nationwide rollout of standardised universal GDM screening. Increased GDM burden raises risks of macrosomia, shoulder dystocia, neonatal birth trauma, and long-term offspring metabolic disorders, imposing heavy clinical and socioeconomic burdens [22,23]. Therefore, optimising pre-pregnancy counselling, antenatal nutritional intervention, regular blood glucose surveillance, and standardised postpartum follow-up is essential to improve perinatal care and gestational metabolic management.

### Temporal trends *vs.* policy effects: challenges in confounding variable control

The major limitation in the interpretation of results is the inextricable confounding between policy periods and secular changes in clinical practices, diagnostic criteria, medical technologies, and the healthcare-seeking behaviours of patients from 2011 to 2025. A series of healthcare system changes occurred independent of fertility policies, including an increase in the emergency obstetric admission rate from 11.9 to 45.3%, a decrease in the average duration of hospital stays from 5.82 days to 4.73 days, and a rise in the ART intervention rate (1.3–6.2%). Without policy-free control cohorts or counterfactual analysis, attributing clinical outcome changes merely to fertility policy reforms is scientifically unreliable. Hence, all detected associations were temporal correlations rather than policy-driven causal effects.

The U-shaped GDM prevalence trend is a typical manifestation of such confounding bias. The decrease in the GDM rate during the initial TCP period was inconsistent with the updated national IADPSG diagnostic criteria, which were expected to enhance diagnostic positivity. Additionally, the main factor contributing to underdiagnosis was the inconsistent universal OGTT screening during 2016–2018. Full-scale universal screening after 2019 and sustained implementation in the UCP period restored authentic GDM epidemiological data. These findings demonstrate that GDM temporal variations were determined by screening and diagnostic protocols rather than fertility policies. Therefore, GDM was not included as a covariate in the multivariate regression models, and its temporal trends were reported as documented prevalence with specific interpretation notes.

### Delivery mode transition and obstetric healthcare system transformation

The hospital caesarean section rate rose steadily from 29.6 to 39.9%, exceeding the global average and aligning with the levels observed in the national trends [9]. Multivariate analysis revealed divergent caesarean section risk changes across policy periods: non-indicated caesarean section control and vaginal birth after caesarean (VBAC) promotion policies constrained caesarean section growth in the TCP period [24], while AMA accumulation, higher gestational complication incidence, and growing scar uterus indications (53.5% in AMA parturients) triggered caesarean section rebound in the UCP period. Scar uterus has become the leading caesarean section indication in China [25,26], indicating that prioritising primiparous vaginal delivery and standardised VBAC management may break the repeated caesarean section cycle [27].

The emergency obstetric admission rate surged from 11.9 to 45.3%, outpacing the growth of routine obstetric service demand. This trend was correlated with expanded high-risk pregnancy volume, aggravated maternal medical anxiety, and insufficient grassroots hospital referral capacity, which is consistent with recent domestic multicentre evidence [28], and partially explained the shortened average hospital stay. Although accelerated inpatient bed turnover relieved ward overcrowding, its potential adverse impacts on obstetric safety require further validation.

### Direct independent effects of fertility policy periods

To avoid overadjustment bias, multivariate regression models eliminated intermediate mediators, including maternal age, parity, AMA status, primipara proportion, scar uterus history, GDM, and HDP. The aORs of policy periods represented direct policy effects excluding mediator-mediated pathways, while total policy effects were presented (Table S3 in the **Online Supplementary Document**). The TCP period was correlated with the highest PPH risk (aOR = 1.80). The elevated PPH risk was associated with policy-relaxation-induced growth of high-risk multiparous parturients and imperfect early-stage management for complicated pregnancies, which is consistent with national PPH epidemiological features [8,29–32].

This study found a negative correlation between caesarean section and PPH with an aOR of 0.28, which contradicts findings from earlier research. This discrepancy does not indicate a clinical protective effect; rather, it highlights measurement bias due to inconsistent diagnostic thresholds for PPH. The cutoff for blood loss to diagnose PPH after a CS (≥1000 mL) is twice that for vaginal deliveries (≥500 mL), which leads to an underestimation of PPH occurrences in the CS group. This definitional bias resulted in a false protective association; consequently, this outcome cannot substantiate the claim that CS reduces PPH risk. Uniform PPH diagnostic criteria or delivery-mode-stratified analyses are recommended for future studies to eliminate this bias.

### Strengths and limitations

This study has notable strengths, including a long observation period, a large sample size, comprehensive clinical data, and the examination of three consecutive fertility policy stages, thereby supporting rigorous longitudinal analysis. Nonetheless, several inherent limitations of this research must be recognised. First, this study relies on single-centre data from a tertiary hospital primarily admitting referred high-risk gravidas. Compared with the nationwide general pregnant population, our cohort carries elevated risks of obstetric complications and emergency hospitalisations. Geographic gaps in medical resources and socioeconomic status inevitably generate selection bias, restricting the external generalisability of our conclusions. Second, the retrospective study design prevents adjustment for uncollected confounders, such as maternal body mass index, detailed surgical histories, and individual socioeconomic backgrounds. Additionally, we only assessed in-hospital maternal and neonatal endpoints, with no long-term post-discharge follow-up health records available for analysis. Third, the fertility policy rollout coincided with ongoing shifts in obstetric clinical practice, including surging emergency admissions, shorter hospital stays, and rising utilisation of IVF, as well as revised diagnostic, admission, and screening standards. Without multi-regional control cohorts and counterfactual statistical modelling, we cannot statistically disentangle secular healthcare and diagnostic trends from policy-specific effects, resulting in ambiguous causal inferences. Evolving national referral pathways and a growing burden of high-risk pregnancies further interfere with intertemporal comparisons of pregnancy outcomes. Moreover, updated standards for diagnosis, screening, and ward management create artificial fluctuations in documented GDM prevalence and maternal ICU admission rates, independent of true shifts in disease severity. The lack of population-level administrative data also precludes quantitative sensitivity analyses to assess bias stemming from these measurement variations. Fourth, comparison of PPH risk across delivery modes is compromised by two distinct biases. Differential blood loss cutoffs for PPH diagnosis between vaginal and caesarean births introduce definitional measurement bias. Moreover, the vaginal delivery cases at this tertiary referral centre are skewed towards unplanned emergency high-risk patients, who have a higher baseline risk for PPH. The failure to fully capture relevant risk modifiers in regression models results in residual indication confounding, thereby distorting the intergroup effect estimates. Fifth, uneven implementation of the OGTT for GDM screening in the early policy era led to underdiagnosis, rendering cross-period comparisons of GDM prevalence invalid. Early-stage incomplete documentation of episiotomy and instrumental vaginal delivery also forced the exclusion of these two indicators from primary analyses; zero recorded cases do not reflect their actual clinical incidence during this timeframe. Sixth, multiple potential mediating variables (maternal age, parity, uterine scarring, GDM, and HDP) were removed from multivariate regression models to mitigate overadjustment. Consequently, our current models only capture the direct impacts of fertility policies, omitting their total mediated effects.

To address the above drawbacks, future work should incorporate mediation analysis to differentiate direct and indirect policy effects fully. We will also collect multicentre data that spans regions with different timelines for the implementation of fertility policies, and adopt counterfactual statistical frameworks. This improved study design will separate long-term clinical secular trends from policy interventions, allowing precise quantification of the net independent effect of fertility policies on obstetric outcomes.

## CONCLUSIONS

Over the past 15 years, China's fertility policies have shifted from birth restriction to progressive childbearing encouragement, a timeframe coinciding with dramatic demographic changes among childbearing women. Based on rigorous clinical data, contemporary pregnant cohorts are featured by AMA, elevated parity, widespread ART conception, and high prior caesarean delivery rates. Such observed shifts in maternal demographics may contribute to rising risks of GDM, placental disorders, peripartum haemorrhage, and other obstetric complications alongside climbing overall caesarean rates, and may partly account for dual pressures on obstetric services driven by surging emergent obstetric demands and shortening average hospital stays. Accordingly, the traditional obstetric system originally designed for young nulliparous women appears poorly matched to the current high-risk pregnancy-dominated clinical setting amid ongoing fertility policy adjustments.

Given that the study originates from a single tertiary referral centre in Guangxi with inherent selection bias toward concentrated high-risk transferred pregnancies, the derived policy suggestions are only fit for optimising local tertiary obstetric services rather than nationwide obstetric system reform. To advance the national fertility promotion strategy, optimise population structure and safeguard maternal and neonatal safety, targeted improvements are proposed from four dimensions:

• construction of refined tiered high-risk pregnancy management and regional cross-hospital referral networks [33];

• popularisation of multidisciplinary team (MDT) mode to boost emergency rescue capacity for PAS and severe PPH;

• the strengthening of public health education to advocate for appropriate-age childbirth and spontaneous vaginal delivery, as well as improving supporting policies to standardise VBAC practice;

• sustaining long-term surveillance on health outcomes linked to evolving fertility policies to generate evidence for targeted clinical interventions [34,35].

Notably, single-centre findings from a single centre cannot serve as a foundation for the development of nationwide obstetric policies; validation through multi-regional multicentre studies is required before any nationwide application of these proposals. In short, multidimensional collaborative improvement and customised obstetric diagnosis-care systems matching current maternal demographic characteristics are likely helpful to secure maternal and infant safety while fulfilling national population development goals.

## Additional material


Online Supplementary Document

## Data Availability

**Data availability:** The data that support the findings of this study are available from the corresponding author upon reasonable request.
