# Supplementary material for: Maternal and perinatal outcomes across China's one-child, two-child, and three-child policy periods: a retrospective cohort analysis of 43,439 deliveries
Source: J Glob Health. 2026 Sep 18;16:04328. doi: 10.7189/jogh.16.04328 (PMC13587160; doi:10.7189/jogh.16.04328)
Supplement: Supplementary file 1 [file jogh-16-04328-s001.pdf]

Supplement to: Haijuan Li, Dongmei Tang, Dan Yang. Maternal and perinatal outcomes across China's one-child, two-child, and three-child policy periods: a retrospective cohort analysis of 43,439 deliveries. J Glob Health. 2026;16:04328.

Table S1. GDM diagnostic criteria, screening protocols, and estimated OGTT coverage across policy periods
Table S2. Interaction tests between policy period and AMA status for cesarean section and PPH
Table S3. Crude (unadjusted) odds ratios for cesarean section and PPH by policy period (total effects, unadjusted for covariates)
Figure S1. Flow chart of participant selection

Table S1. GDM diagnostic criteria, screening protocols, and estimated OGTT coverage across policy periods

Policy period	Diagnostic criteria	Screening protocol	Estimated OGTT coverage	Recorded GDM prevalence	Notes
OCP (2011–2015)	2010 IADPSG (selective)	Risk-factor based + 50 g GCT; 75 g OGTT only if GCT positive	~40–50%	10.2%	Selective screening; likely missed cases
TCP (2016–2020)	2010 IADPSG (universal)	75 g OGTT recommended for all at 24–28 weeks	2016–2018: ~30–40%; 2019–2020: ~80–90%	2.6%	Early TCP period had low OGTT coverage; underdiagnosis likely
UCP (2021–2025)	2010 IADPSG (universal)	75 g OGTT universally applied at 24–28 weeks	~95%	26.7%	Consistent universal screening; most reliable estimate

GCT – glucose challenge test, GDM – gestational diabetes mellitus, IADPSG – International Association of Diabetes and Pregnancy Study Groups, OCP – one-child policy, OGTT – oral glucose tolerance test, TCP – two-child policy, UCP – universal three-child policy.

Table S2. Interaction tests between policy period and AMA status for cesarean section and PPH

Outcome	Comparison	*P* for interaction	Adjusted for
Cesarean section	TCP vs. OCP × AMA	0.18	Twin pregnancy, placenta previa, macrosomia, preterm birth
	UCP vs. OCP × AMA	0.24	Twin pregnancy, placenta previa, macrosomia, preterm birth
PPH	TCP vs. OCP × AMA	0.32	Twin pregnancy, placenta previa, macrosomia, preterm birth, mode of delivery
	UCP vs. OCP × AMA	0.41	Twin pregnancy, placenta previa, macrosomia, preterm birth, mode of delivery

AMA – advanced maternal age, OCP – one-child policy, PPH – postpartum hemorrhage, TCP – two-child policy, UCP – universal three-child policy.

Table S3. Crude (unadjusted) odds ratios for cesarean section and PPH by policy period (total effects, unadjusted for covariates)

Outcome	Policy period	Crude OR	95% CI	*P* value
Cesarean section	TCP vs. OCP	1.11	1.06–1.16	<0.001
	UCP vs. OCP	1.35	1.28–1.42	<0.001
PPH	TCP vs. OCP	2.04	1.77–2.36	<0.001
	UCP vs. OCP	1.45	1.22–1.73	<0.001

Crude OR = (Odds of outcome in policy period)/(Odds of outcome in OCP period), unadjusted for covariates. CI – confidence interval, OCP – one-child policy, OR – odds ratio, PPH – postpartum hemorrhage, TCP – two-child policy, UCP – universal three-child policy.

Figure S1. Flow chart of participant selection

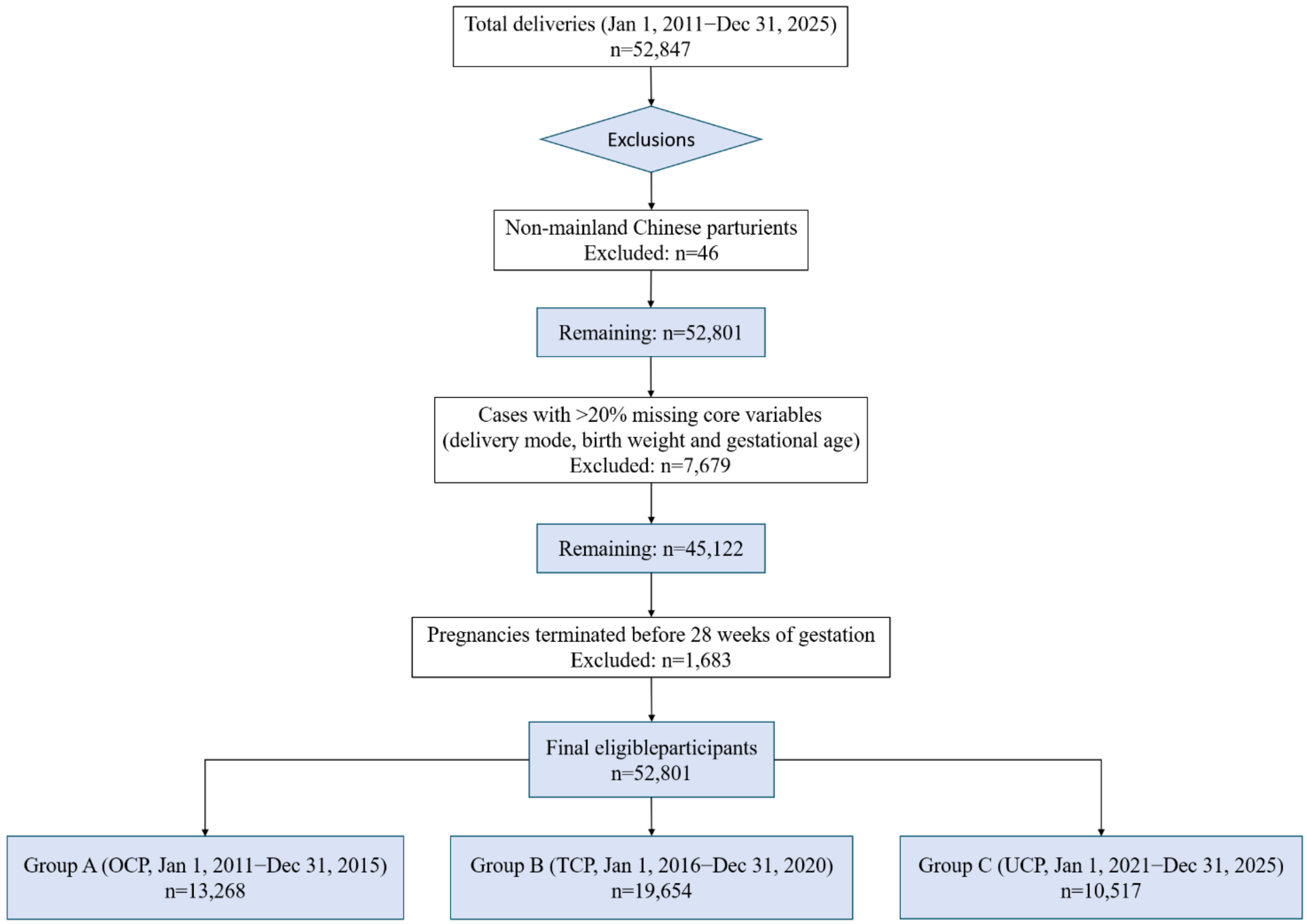

OCP – one-child policy, TCP – two-child policy, UCP – universal three-child policy.
